# Deconvolution of the Functional Ultrasound Response in the Mouse Visual Pathway Using Block-Term Decomposition

**DOI:** 10.1007/s12021-022-09613-3

**Published:** 2022-11-15

**Authors:** Aybüke Erol, Chagajeg Soloukey, Bastian Generowicz, Nikki van Dorp, Sebastiaan Koekkoek, Pieter Kruizinga, Borbála Hunyadi

**Affiliations:** 1 Circuits and Systems (CAS), Department of Microelectronics, Delft University of Technology, Mekelweg 5, Delft, 2628 CD The Netherlands; 2 Center for Ultrasound and Brain imaging at Erasmus MC (CUBE), Department of Neuroscience, Erasmus Medical Center, Doctor Molewaterplein 40, Rotterdam, 3015 GD The Netherlands

**Keywords:** Hemodynamic response function, Deconvolution, Functional ultrasound, Fensor decomposition, Mouse, Visual perception

## Abstract

Functional ultrasound (fUS) indirectly measures brain activity by detecting changes in cerebral blood volume following neural activation. Conventional approaches model such functional neuroimaging data as the convolution between an impulse response, known as the hemodynamic response function (HRF), and a binarized representation of the input signal based on the stimulus onsets, the so-called experimental paradigm (EP). However, the EP may not characterize the whole complexity of the activity-inducing signals that evoke the hemodynamic changes. Furthermore, the HRF is known to vary across brain areas and stimuli. To achieve an adaptable framework that can capture such dynamics of the brain function, we model the multivariate fUS time-series as convolutive mixtures and apply block-term decomposition on a set of lagged fUS autocorrelation matrices, revealing both the region-specific HRFs and the source signals that induce the hemodynamic responses. We test our approach on two mouse-based fUS experiments. In the first experiment, we present a single type of visual stimulus to the mouse, and deconvolve the fUS signal measured within the mouse brain’s lateral geniculate nucleus, superior colliculus and visual cortex. We show that the proposed method is able to recover back the time instants at which the stimulus was displayed, and we validate the estimated region-specific HRFs based on prior studies. In the second experiment, we alter the location of the visual stimulus displayed to the mouse, and aim at differentiating the various stimulus locations over time by identifying them as separate sources.

## Introduction

Functional ultrasound (fUS) is a relatively new neuroimaging technique that that captures changes in local blood dynamics. More precisely, fUS makes use of plane waves transmitted at an ultrafast frame rate that are backscattered by the moving red blood cells in the imaged area. Thus, the fUS signal amplitude fluctuates over time in proportion to alterations in local blood volume (Deffieux et al., 2021). The hemodynamic activity as detected by fUS can be used as an indirect report of changing neural activity through the phenomenon of neurovascular coupling (Aydin et al., 2020). In particular, local variations in neural activity induce a delayed response in blood flow and volume known as functional hyperemia, which can be modelled by a hemodynamic response function of time. The sensitivity of fUS in measuring subtle variations of blood dynamics has led to a variety of fUS-based animal and clinical studies in the past decade, ranging from detection of responses to sensory stimuli to complex brain states and behavior (Nunez-Elizalde et al., 2022). These include studies on small rodents (Macé et al., 2011; Gesnik et al., 2017; Macé et al., 2018; Koekkoek et al., 2018), nonhuman primates (Blaize et al., 2020; Norman et al., 2021), birds (Rau et al., 2018) and humans (Imbault et al., 2017; Baranger et al., 2021; Soloukey et al., 2020).

Understanding the underlying mechanisms of hemodynamic response has been an important challenge not only for fUS (Nunez-Elizalde et al., 2022), but also for several other established functional neuroimaging modalities, including functional magnetic resonance imaging (fMRI) (Schrouff et al., 2013; Roels et al., 2015) and functional near-infrared spectroscopy (fNIRS) (Shah & Seghouane, 2014)). A common way to model the measured time-series is via a function representing the impulse response of the neurovascular system, known as the hemodynamic response function (HRF) (Seghouane & Shah, 2012). In this model, the HRF gets convolved with an input signal representing the experimental paradigm (EP), which is expressed as a binary vector that shows the on- and off- times of a given stimulus. However, not all stimuli can be predefined, i.e. under the experimenters’ control (Karahanoglu et al., 2013). For instance, brain reaction to mental imagery is shown to be almost as strong as the activity evoked by real perception in certain brain regions under a variety of experimental designs, such as visual (Ganis et al., 2004) or auditory (Bunzeck et al., 2005). Therefore, the input signals that represent such tasks or events that evoke brain activity should be generalized beyond merely the preset paradigms. This issue has been addressed by (Karahanoglu et al., 2013; Karahanoglu & Van De Ville, 2015; Uruñuela et al., 2021), where the authors have defined the term *activity-inducing* signal, which, as the name suggests, comprises any input signal that induces hemodynamic activity. We will refer to activity-inducing signals as *source signals* in the rest of this paper, which steers the reader to broader terminology not only used in biomedical signal processing, but also in acoustics and telecommunications (Naik & Wang, 2014), and emphasizes that recorded output data are *sourced* by such signals.

An accurate estimation of the HRF is crucial to correctly interpret both the hemodynamic activity itself and the underlying source signals. Furthermore, the HRF has shown potential as a biomarker for healthy aging (West et al., 2019) or pathological brain functioning; examples of which include obsessive-compulsive disorder (Rangaprakash et al., 2021), mild traumatic brain injury (Mayer et al., 2014), Alzheimer’s disease (Asemani et al., 2017), epilepsy (Van Eyndhoven et al., 2021) and severe psychosocial stress (Elbau et al., 2018). While HRFs can as well be defined in nonlinear and dynamic frameworks with the help of Volterra kernels (Friston, 2002), linear models have particularly gained popularity due to the combination of their remarkable performance and simplicity. Several approaches have been proposed in the literature which employ linear modelling for estimating the HRF. The strictest approach assumes a constant a priori shape of the HRF, i.e. a mathematical function with fixed parameters, and is only concerned with finding its scaling (the activation level). The shape used in this approach is usually given by the canonical HRF model (Friston et al., 1998). As such, this approach does not incorporate HRF variability, yet the HRF is known to change significantly across subjects, brain regions and triggering events (Handwerker et al., 2004; Aguirre et al., 1998; Fransson et al., 1999). A second approach is to estimate the parameters of the chosen shape function, which leads to a more flexible solution (Aydin et al., 2020). Alternatively, HRF estimation can be reformulated as a regression problem by expressing the HRF as a linear combination of several basis functions (which are often chosen to be the canonical HRF and its derivatives). This approach is known as the general linear model (GLM) (Lindquist et al., 2009). Finally, it is also possible to apply no shape constraints on the HRF, and predict the value of the HRF distinctly at each time point. This approach suffers from high computational complexity and variance of the estimated HRFs, which might be of arbitrary or physiologically meaningless forms (Glover, 1999).

Note that the majority of studies which tackle HRF estimation presume that the source signal is known and equal to the EP, leaving only one unknown in the convolution: the HRF (Hütel et al., 2021). However, as mentioned earlier, a functional brain response can be triggered by more sources than the EP alone. These sources can be extrinsic, i.e., related to environmental events, such as unintended background stimulation or noise artefacts. They might also be intrinsic sources, such as mental imagery. Under such complex and multi-causal circumstances, recovering the rather ’hidden’ source signal(s) can be of interest. Moreover, even the EP itself can be much more complex than what a simple binary pattern allows for. Indeed, the hemodynamic response to, for instance, a visual stimulus, can vary greatly depending on its parameters, such as its contrast (Gesnik et al., 2017), demanding a continuous variable to represent the “on” times of the stimulus. In contrast to the aforementioned methods, where the goal was to estimate HRFs from a known source signal, there have also been attempts to predict the sources by assuming a known and fixed HRF (Caballero et al., 2011) (Karahanoglu et al., 2013). However, these methods fall short of depicting the HRF variability.

To sum up, neither the sources nor the HRF are straightforward to model, and as such, when either is assumed to be fixed, it can easily lead to misspecification of the other. Therefore, we consider the problem of jointly estimating the source signals and HRFs from multivariate fUS time-series. This problem has been addressed by (Sreenivasan et al., 2015), (Wu et al., 2013), (Cherkaoui et al., 2019) and (Cherkaoui et al., 2021). In (Sreenivasan et al., 2015), it is assumed that the source signal (here considered as neural activity) lies in a high frequency band compared to the HRF, and can thus be recovered using homomorphic filtering. On the other hand, (Wu et al., 2013) first estimates a spike-like source signal by thresholding the fMRI data and selecting the time points where the response begins, and subsequently fits a GLM using the estimated source signal to find the HRF. Both of the aforementioned methods are univariate: although they analyze multiple regions and/or subjects, the analysis is performed separately on each time series, thereby ignoring any mutual information shared amongst biologically relevant ROIs.

Recently, a multivariate deconvolution of fMRI time series has been proposed in (Cherkaoui et al., 2021). The authors proposed an fMRI signal model, where neural activation is represented as a low-rank matrix - constructed by a certain (low) number of temporal activation patterns and corresponding spatial maps encoding functional networks - and the neural activation is linked with the observed fMRI signals via region-specific HRFs. The main advantage of this approach is that it allows whole-brain estimation of HRF and neural activation. However, all HRFs are defined via the dilation of a presumed shape, which may not be enough to capture all possible variations of the HRF, as the width and peak latency of the HRF are coupled into a single parameter. Moreover, the estimated HRFs are region-specific, but not source-specific. Therefore, the model cannot account for variations in the HRF due to varying stimulus properties. Yet, the length and intensity of stimuli appear to have a significant effect on HRF shape even within the same region, as observed in recent fast fMRI studies (Chen et al., 2021).

In order to account for the possible variations of the HRF for both different sources and regions, we model the fUS signal in the framework of convolutive mixtures, where multiple input signals (sources) are related to multiple observations (measurements from a brain region) via convolutive mixing filters. In the context of fUS, the convolutive mixing filters stand for the HRFs, which are unique for each possible combination of sources and regions, allowing variability across different brain areas and triggering events. In order to improve identifiability, we make certain assumptions, namely that the shape of the HRFs can be parametrized and that the source signals are uncorrelated. Considering the flexibility of tensor-based formulations for the purpose of representing such structures and constraints that exist in different modes or factors of data (Sorber et al., 2015), we solve the deconvolution by applying block-term decomposition (BTD) on the tensor of lagged measurement autocorrelation matrices.

While in our previous work (Erol et al., 2020) we had considered a similar BTD-based deconvolution, this paper presents several novel contributions. First, we improve the robustness of the algorithm via additional constraints and a more sophisticated selection procedure for the final solution from multiple optimization runs. We also present a more detailed simulation study considering a large range of possible HRF shapes. Finally, we demonstrate the capabilities of our method on two experimental datasets recorded from mice using fUS during visual stimulation. In the first dataset we track the visual information pathway by investigating the peak latency of the HRF in key anatomical structures involved within the mouse brain’s colliculo-cortical, image-forming visual pathway: the lateral geniculate nucleus (LGN), the superior colliculus (SC) and the primary visual cortex (V1). We show that the ordering of the peak latencies agrees with prior works (Gesnik et al., 2017), confirming with fUS that visual information first travels through the subcortical targets SC and LGN, before being relayed to V1. In the second experiment we repeatedly display the visual stimuli at 5 distinct locations. We show that our technique is able to extract 5 underlying sources, and the time course of each of these sources have a one-to-one correspondence with the timing of the 5 distinct stimulus locations.

The rest of this paper is organized as follows. First, we describe our data model and the proposed tensor-based solution for deconvolution. Next, we describe the experimental setup and data acquisition steps used for fUS imaging of a mouse subject. This is followed by the deconvolution results, which are presented in two-folds: *(i)* Numerical simulations, and *(ii)* Results on real fUS data. Next, under discussion, we review the highlights of our modelling and results, and elaborate on the neuroscientific relevance of our findings. Finally, we state several future extensions and conclude our paper.

## Signal Model

Naturally, fUS images contain far more pixels than the number of anatomical or functional regions. We therefore expect certain groups of pixels to show similar signal fluctuations along time, and we consider the fUS images as parcellated in space into several regions. Consequently, we represent the overall fUS data as an $M \times N$ matrix, where each of the *M* rows contain the average pixel time-series within a region-of-interest (ROI), and *N* is the number of time samples.

Assuming a single source signal, a single ROI time-series *y*(*t*) can be written as the convolution between the HRF *h*(*t*) and the input source signal *s*(*t*) as:

1 $$\begin{aligned} y(t) = \sum _{l=0}^L h(l)s(t-l) \end{aligned}$$

where $L+1$ gives the HRF filter length. However, a single ROI time-series may be affected by a number of (*R*) different source signals. Each source signal $s_r(t)$ may elicit a different HRF, $h_r(t)$. Therefore, the observed time-series is the summation of the effect of all underlying sources:

2 $$\begin{aligned} y(t) = \sum _{r=1}^R \sum _{l=0}^L h_r(l)s_r(t-l). \end{aligned}$$

Finally, extending our model to multiple ROIs, where each ROI may have a different HRF, we arrive to the multivariate convolutive mixture formulation:

3 $$\begin{aligned} y_m(t) = \sum _{r=1}^R \sum _{l=0}^L h_{mr}(l)s_r(t-l) \end{aligned}$$

where $h_{mr}(l)$ is the convolutive mixing filter, belonging to the ROI *m* and source *r* (Mitianoudis & Davies, 2003). Note that, in this work we consider that the ROIs are known (for instance, via anatomical labelling (Wu et al., 2013)), or can be estimated from the data as a pre-processing step prior to deconvolution. We follow the latter approach in this paper, and apply independent component analysis (ICA) on the fUS data, regarding which more details will be explained in “Experimental Setup and Data Acquisition”.

In the context of fUS, the sources that lead to the time-series can be task-related (*T*), such as the EP, or artifact-related (*A*). The task-related sources are convolved with an HRF, whereas the artifact-related sources are directly additive on the measured time-series (Marrelec et al., 2003). Artifact sources in general are used to represent fUS signal variation of non-neural origin. Under this definition, we consider physiological noise, e.g. movement of the subject causing signal fluctuations in the entire field-of-view. Moreover, a recent study (Nunez-Elizalde et al., 2022) found out that only the low-frequency content of the fUS signal reflects neural activity. Artifact sources can as well incorporate instrumentation noise, such as thermal or electronic noise (commonly modeled as additive, (Demené et al., 2015a; Huang et al., 2019)) introduced by the ultrasound acquisition system, which can be spatially varying, becoming more prominent at deeper areas of the brain. As such, the strength of the effect that an artifact source exerts on a region should depend on the artifact type and the brain region. Hence, each $h_{mr}(l)$ with $r \in A$ should correspond to a scaled (by $a_{mr}$) unit impulse function (ensuring additivity). Finally, we rewrite Eq. 3 as:

4 $$\begin{aligned} \begin{aligned} y_m(t)&= \sum _{r\in T} \sum _{l=0}^L h_{mr}(l)s_r(t-l)+\sum _{r\in A} \sum _{l=0}^{L} a_{mr} \delta (l)s_r(t-l) \\&= \sum _{r\in T} \sum _{l=0}^L h_{mr}(l)s_r(t-l)+\sum _{r\in A} a_{mr} s_r(t). \end{aligned} \end{aligned}$$

We aim at solving this deconvolution problem to recover the sources and HRFs of interest separately at each ROI *m*.

## Proposed Method

In this section, we will present the steps of the proposed tensor-based deconvolution method. We will first introduce how deconvolution of the observations modeled as in Eq. 4 can be expressed as a BTD. Due to the fact that this problem is highly non-convex, we will subsequently explain our approach to identifying a final solution for the decomposition. Finally, we will describe source signal estimation using the HRFs predicted by BTD.

### Formulating the Block-Term Decomposition

We start by expressing the convolutive mixtures formulation in matrix form. First, the output time-series and source signals are re-organized into block-Hankel matrices $\mathbf {Y}$ and $\mathbf {S}$ of size $ML'\times (N-L')$ and $R(L+L')\times (N-(L+L'))$ respectively. More specifically, the columns of $\mathbf {Y}$ and $\mathbf {S}$ contain (lagged versions of) the output and source signals, denoted by $\mathbf {y}(n)$, $n=1,\dots ,N-L'$ and $\mathbf {s}(n)$, $n=1,\dots ,N-(L+L')$ respectively. Here, the parameter $L'$ controls the size of the tensor of lagged output autocorrelations to be decomposed, regarding which more details will follow later within this section. The column vectors $\mathbf {y}(n)$ and $\mathbf {s}(n)$ are constructed as follows (Bousbia-Salah et al., 2001):

5 $$\begin{aligned} \begin{aligned} \mathbf {y}(n)&= [y_1(n),...,y_1(n-L'+1), \\&...,y_M(n),...,y_M(n-L'+1)]^T\; \; \text {and}\\ \mathbf {s}(n)&= [s_1(n),...,s_1(n-(L+L')+1), \\&...,s_R(n),...,s_R(n-(L+L')+1)]^T. \end{aligned} \end{aligned}$$

This way, Eq. 3 can be written as $\mathbf {Y}=\mathbf {H}\mathbf {S}$, where $\mathbf {H}$ is the mixing matrix containing the convolutive mixing filters in the form of Toeplitz matrices:

6 $$\begin{aligned} \mathbf {H}=[\mathbf {H}_1 \quad \dots \quad \mathbf {H}_R]= \begin{bmatrix} \mathbf {H_{11}} &{} \dots &{} \mathbf {H_{1R}}\\ \vdots &{} \ddots &{} \vdots \\ \mathbf {H_{M1}} &{} \dots &{} \mathbf {H_{MR}} \end{bmatrix} \end{aligned}$$

whose any block-entry $\mathbf {H}_{mr}$ is the Toeplitz matrix of $h_{mr}(l)$:

7 $$\begin{aligned} \mathbf {H}_{mr}= \begin{bmatrix} h_{mr}(0) &{} \dots &{} h_{mr}(L) &{} \dots &{} 0\\ &{} \ddots &{} \ddots &{} \ddots &{} \\ 0 &{} \dots &{} h_{mr}(0) &{} \dots &{} h_{mr}(L) \end{bmatrix} . \end{aligned}$$

Next, the autocorrelation $\mathbf {R}_{\mathbf {y}}(\tau )$ for a time lag $\tau$ is expressed as:

8 $$\begin{aligned} \mathbf {R}_{\mathbf {y}}(\tau )&= \mathrm {E}\{\mathbf {y}(n)\mathbf {y}(n+\tau )^T\} = \mathrm {E}\{\mathbf {H}\mathbf {s}(n)\mathbf {s}(n+\tau )^T\mathbf {H}^T\} \nonumber \\&=\mathbf {H} \mathbf {R}_{\mathbf {s}}(\tau )\mathbf {H}^T, \; \; \; \; \forall \tau . \end{aligned}$$

**Fig. 1:**
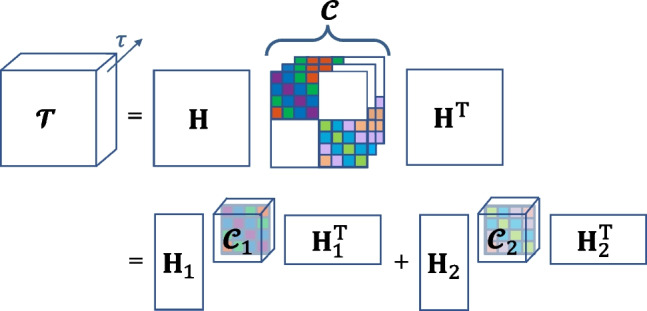
Demonstration of BTD for $R=2$. The tensor $\varvec{\mathcal {T}}$ of stacked output autocorrelations $\mathbf {R}_\mathbf {y}(\tau )$, $\forall \tau$ is first expressed in terms of $\mathbf {H}$ and a core tensor $\varvec{\mathcal {C}}$, which stores the stacked source autocorrelations $\mathbf {R}_\mathbf {s}(\tau )$, $\forall \tau$. Each $\mathbf {R}_\mathbf {s}(\tau )$ corresponds to a frontal slice of $\varvec{\mathcal {C}}$ and exhibits a block-diagonal structure with inner Toeplitz-blocks. Note that, each slice comes as a lagged version of the preceeding slice. $\varvec{\mathcal {T}}$ is decomposed into *R* terms, each of which contains a core tensor ($\varvec{\mathcal {C}}_1$ or $\varvec{\mathcal {C}}_2$, representing the autocorrelation of the corresponding source) and a block column of $\mathbf {H}$ ($\mathbf {H}_1$ or $\mathbf {H}_2$)

Notice that $L'$ determines the number of variables (per region) for computing the autocorrelations $\mathbf {R}_{\mathbf {y}}(\tau )$ of size $ML'\times ML'$, whereas $N-L'$, the number of columns of $\mathbf {Y}$, corresponds to the number of observations. Assuming that the sources are uncorrelated, the matrices $\mathbf {R}_\mathbf {s}(\tau )$ are block-diagonal, i.e. non-block-diagonal terms representing the correlations between different sources are 0. Therefore, the output autocorrelation matrix $\mathbf {R}_\mathbf {y}(\tau )$ is written as the block-diagonal matrix $\mathbf {R}_\mathbf {s}(\tau )$ multiplied by the mixing matrix $\mathbf {H}$ from the left and by $\mathbf {H}^\text {T}$ from the right. Then, stacking the set of output autocorrelation matrices $\mathbf {R}_\mathbf {y}(\tau )$ for *K* different values of $\tau$ will give rise to a tensor $\varvec{\mathcal {T}}$ of size $ML'\times ML'\times K$ that admits a so-called block-term decomposition (BTD). Eventually, $\varvec{\mathcal {T}}$ can be written as a sum of low-multilinear rank tensors, in this specific case a rank of $(L+L',L+L',\cdot )$ (Van Eeghem & De Lathauwer, 2017). The decomposition for $R=2$ is illustrated in Fig. 1.

Due to the Hankel-block structure of $\mathbf {Y}$ and $\mathbf {S}$, $\mathbf {R}_{\mathbf {y}}(\tau )$ and $\mathbf {R}_{\mathbf {s}}(\tau )$ are Toeplitz-block matrices. Furthermore, as each frontal slice of $\varvec{\mathcal {T}}$ is constructed using a different time-lag $\tau$ in $\mathbf {R}_{\mathbf {y}}(\tau )$, the slices come as shifted versions of one another (the same shift-structure is valid for $\varvec{\mathcal {C}}$, constructed with $\mathbf {R}_{\mathbf {s}}(\tau )$’s, as shown in Fig. 1). As such, the construction of each whole core tensor ($\varvec{\mathcal {C}}_r$ for the *r*th source) is based on a single vector, $\mathbf {z}_r$. We will denote the aforementioned transformations (first the formation of a Toeplitz matrix out of $\mathbf {z}_r$, and later shifting this Toeplitz matrix at various lags such that they are placed at different slices of a tensor) using the operator $\lambda$, such that $\varvec{\mathcal {C}}_r=\lambda (\mathbf {z}_r)$. Note that the number of time-lags to be included is a hyperparameter of the algorithm, and we take it as equal to the filter length in this work.

Considering our signal model, where we have defined two types of sources, we can rewrite the block-columns of $\mathbf {H}=[\mathbf {H}_1 \; \mathbf {H}_2]$ (Eq. 6) simply as $\mathbf {H}=[\mathbf {H}_T \; \mathbf {H}_A]$. Here, $\mathbf {H}_T$ relates to the task-sources, i.e. includes the region-specific HRFs, whereas $\mathbf {H}_A$ includes the region-specific scalings of the artifact sources.

**Fig. 2:**
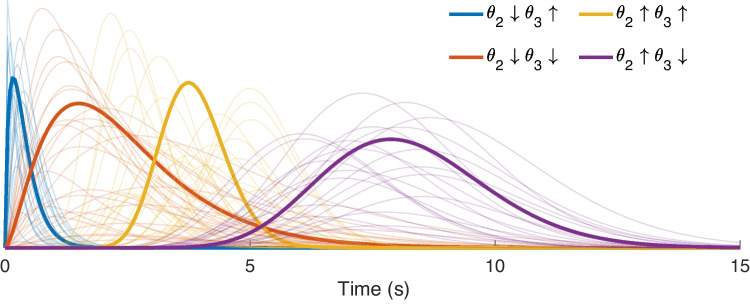
Example HRF shapes constructed using Eq. 9. In order to illustrate the effect of $\theta _2$ and $\theta _3$ individually, we generated HRF shapes using different combinations of “low range” and “high range” values of each parameter. The low range of $\theta _2$ and $\theta _3$ (indicated by the downward arrow in the legends) were both selected randomly in the interval [1, 3], whereas their high range (indicated by the upward arrow in the legends) were selected randomly amongst the intervals [15, 25] and [4, 6] respectively

In addition, we impose a shape constraint to the HRFs such that they are physiologically interpretable. To this end, we adapted the canonical HRF model used predominantly in fMRI studies (Friston et al., 1998) for depicting CBV changes by removing the second gamma function leading to the undershoot response (as similarly applied by Aydin et al. (2020)). Thus, we expresses the HRF in terms of a single gamma function defined on a parameter set $\varvec{\theta }$:

9 $$\begin{aligned} h(t,\varvec{\theta }) = \theta _1(\Gamma (\theta _2)^{-1} \theta _3^{\theta _2}t^{\theta _2-1}\mathrm {e}^{-\theta _3t}) \end{aligned}$$

where $\theta _1$, $\theta _2$ and $\theta _3$ control the response height, delay and dispersion of the HRF respectively. By adjusting these parameters, it is possible to model a wide range of HRF shapes, of which some examples are shown in Fig. 2. In order to preserve this variety in our solutions, we do not apply any bounds or constraints on the HRF parameters except for non-negativity, i.e. $\varvec{\theta }>\varvec{0}$.

Finally, the BTD is computed by minimizing the following cost function:

10 $$\begin{aligned} \begin{aligned} J(\varvec{\mathcal {C}},\varvec{\theta },\mathbf {a})&= \Vert \;\, \varvec{\mathcal {T}}&- \sum _{r \in T} \varvec{\mathcal {C}}_r \times _1 \mathbf {H}_r(\varvec{\theta }_r) \times _2 \mathbf {H}_r(\varvec{\theta }_r) \\&\;\;\;&-\sum _{r \in A} \varvec{\mathcal {C}}_r \times _1 \mathbf {H}_r(\mathbf {a}_r) \times _2 \mathbf {H}_r(\mathbf {a}_r) \Vert ^2_F \\ \text {s.t. }\varvec{\mathcal {C}}_r&= \lambda (\mathbf {z}_r), \\ \varvec{\theta }_r&> \varvec{0} \end{aligned} \end{aligned}$$

where $\mathbf {H}_r$ shows the *r*th block column of $\mathbf {H}$ and $\varvec{\theta }_r$ shows the dependency of $\mathbf {H}_r$ (i.e., regional HRFs assigned to the *r*th source) on its own set of parameters. The operator $||\cdot ||_F$ is the Frobenius norm.

We optimize Eq. 10 using the structured data fusion (SDF) framework, more specifically using the quasi-Newton algorithm sdf_minf, offered by Tensorlab (Vervliet et al., 2016). In order to overcome the sensitivity of this algorithm to initial point selection (Sorber et al., 2013a), we run the BTD several times with random initializations, and use a clustering-based approach to determine the best solution from these runs. In the next section, we will elaborate on our selection procedure.

### Identifying a Stable Solution for BTD

For many matrix and tensor-based factorizations, such as the BTD described above, the objective functions are non-convex. As such, the algorithm selected for solving the non-convex optimization might converge to local optimas of the problem (Luo et al., 2011). In order to identify a stable solution, it is common practice to run the optimization multiple times, with a different initialization at each run. Finally, a choice needs to be made amongst different solutions of the decomposition. Unfortunately, the solution with the lowest cost value is not guaranteed to yield the most meaningful result (Van Eyndhoven et al., 2019).

For our problem, each BTD repetition produces *M* HRFs, characterized by their parameters $\varvec{\theta }_m, m=1,2,\dots ,M$. We follow a similar approach as in the *Icasso* software developed for instantaneous independent component analysis (Himberg et al., 2004), where multiple solutions of the same problem are clustered, revealing that reliable estimates reside in tight clusters, whereas unreliable ones do not belong to any such cluster. Likewise, we use the peak latencies of the estimated HRFs as our features and cluster the BTD solutions. The steps of our clustering approach are as follows:

1. Run BTD 20 times with random initializations, and from each run, store the following:
  Final value of the cost (i.e., objective) function
  *M* HRFs
2. Eliminate the *P* outlier BTD repetitions having significantly higher cost values (We use Matlab’s imbinarize for the elimination which chooses an optimal threshold value based on Otsu’s method (Otsu, 1979), as we expect the best solution to be amongst the low-cost solutions)
3. Form a matrix with *M* columns (standing for the peak latencies of *M* HRFs, these are the features) and $20-P$ rows (standing for the retained BTD repetitions, these are the observations)
4. Apply agglomerative hieararchical clustering to the columns of the matrix in Step 3
5. Compute the following intracluster distance metric for each cluster as:
  11 $$\begin{aligned} d_\text {C} = \frac{\max _{c_1,c_2 \in \text {C}} d(c_1,c_2)}{n_\text {C}} \end{aligned}$$
  where the numerator gives the Euclidean distance between the two most remote observations inside the cluster $\text {C}$ (known as the complete diameter distance (Bolshakova & Azuaje, 2003)), and the denominator, $n_\text {C}$, is the number of observations included in $\text {C}$
6. Determine the most stable cluster as the one having the minimum intracluster distance
7. Compute the mean of the estimated HRFs belonging to the cluster of Step 6

To sum up, the clustering approach described above assumes that the best possible solution will be low-cost (Step 2), have low intracluster distance (numerator of Eq. 11) and frequently-occurring (denominator of Eq. 11). Note that, the average run-time for the BTD of, for instance, a $192 \times 192 \times 32$ tensor (representing the lagged autocorrelation tensor of size $ML'\times ML' \times K$ of the original data matrix obtained from the first fUS experiment with the following parameters: $M=3 \text { regions}, \; N=1430 \text { time points}, \; R=2 \text { sources}, \; L=K=(\text {fUS sampling rate} = 4 \text { Hz})*(8 \text { seconds})=32 \text { samples}, \; L'=64 \text { samples}$) is $\sim 30$ seconds, thus running the BTD 20 times to reach to a final solution leads to a total run-time of 10 minutes.

After computing the final HRF predictions, the last step is to estimate the sources.

### Estimation of the Source Signals

The final HRF estimates are reorganized in a Toeplitz-block matrix as shown in Eqs. 6 and 7. This gives rise to $\hat{\mathbf {H}}_r$’s ($r=1,2,...,R$), i.e., the block columns of $\mathbf {H}$ which contain the estimated convolutive mixing filters that are associated to source *r* at different regions. Going back to our initial formulation $\mathbf {Y}=\mathbf {H}\mathbf {S}$, we can estimate the source signals $\mathbf {S}_r$ by:

12 $$\begin{aligned} \hat{\mathbf {S}}_r=\hat{\mathbf {H}}_r^\dagger \mathbf {Y} \end{aligned}$$

where $(.)^\dagger$ shows the Moore-Penrose pseudo-inverse.

In order to obtain the pseudo-inverse of $\hat{\mathbf {H}}_r$’s, we used truncated singular value decomposition (SVD). Truncated SVD is a method for calculating the pseudo-inverse of a rank-deficient matrix, which is the case for many signal processing applications on real data, such as for extraction of signals from noisy environments (Demmel, 1997). Stabilization of the pseudo-inverse in presence of noise can be achieved by choosing the optimal number of singular values of $\hat{\mathbf {H}}_r$ to be discarded, which can be viewed as a regularization problem (Sano, 1993). In this work, we determine this number heuristically.

## Experimental Setup and Data Acquisition

We used two mice (C57BL/6J in the single-stimulus, and B6CBAF1/JRj in the multiple-stimuli experiment; both 7 months-old and male) for the *in vivo* fUS experiments. The experimental setup depicted in Fig. 4. The mice were housed with food and water *ad libitum*, and maintained under standard conditions (12/12 h light-darkness cycle, 22 $^\circ$C). Preparation of each mice involved surgical pedestal placement and craniotomy. First, an in-house developed titanium pedestal (8 mm in width) was placed on the exposed skull using an initial layer of bonding agent (OptiBond™) and dental cement (Charisma^®^). Subsequently, a surgical craniotomy was performed to expose the cortex from Bregma -1 mm to -7 mm. After skull bone removal and subsequent habituation, the surgically prepared, awake mouse was head-fixed and placed on a movable wheel in front of two stimulation screens (Dell 23,8” S2417DG, 1280 x 720 pixels, 60 Hz) in landscape orientation, positioned at a 45° angle with respect to the antero-posterior axis of the mouse, as well as 20 cm away from the mouse’s eye, similar to (Macé et al., 2018). All experimental procedures were approved *a priori* by an independent animal ethical committee (DEC-Consult, Soest, the Netherlands), and were performed in accordance with the ethical guidelines as required by Dutch law and legislation on animal experimentation, as well as the relevant institutional regulations of Erasmus University Medical Center.

In the first experiment, the visual stimulus consisted of a rectangular patch of randomly generated, high-contrast images - white “speckles” against a black background - which succeeded each other with 25 frames per second, inspired by (Niranjan et al., 2016; Gesnik et al., 2017; Ito et al., 2017). The rectangular patch spanned across both stimulation screens such that it was centralized in front of the mouse, whereas the screens were kept entirely black during the rest (i.e., non-stimulus) periods. The visual stimulus was presented to the mouse in 20 blocks of 4 seconds in duration. Each repetition of the stimulus was followed by a random rest period between 10 to 15 seconds.

**Fig. 3:**
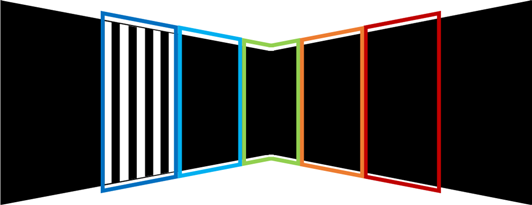
Five locations of the grating stimulus on the stimulation screens

In the second experiment, the visual stimulus was a sinusodial grating presented randomly at one of 5 predefined locations, determined by dividing the mouse horizontal field-of-view ($140^{\circ }$ (Marshel et al., 2011)) as projected on the screens into 5 equal parts. These locations are displayed in Fig. 3. The grating was drifted at 4 degrees per cycle, and at a temporal frequency (cycles per second) of 8.3 Hz. The stimulus and rest duration was kept fixed at 10 and 15 seconds respectively.

**Fig. 4:**
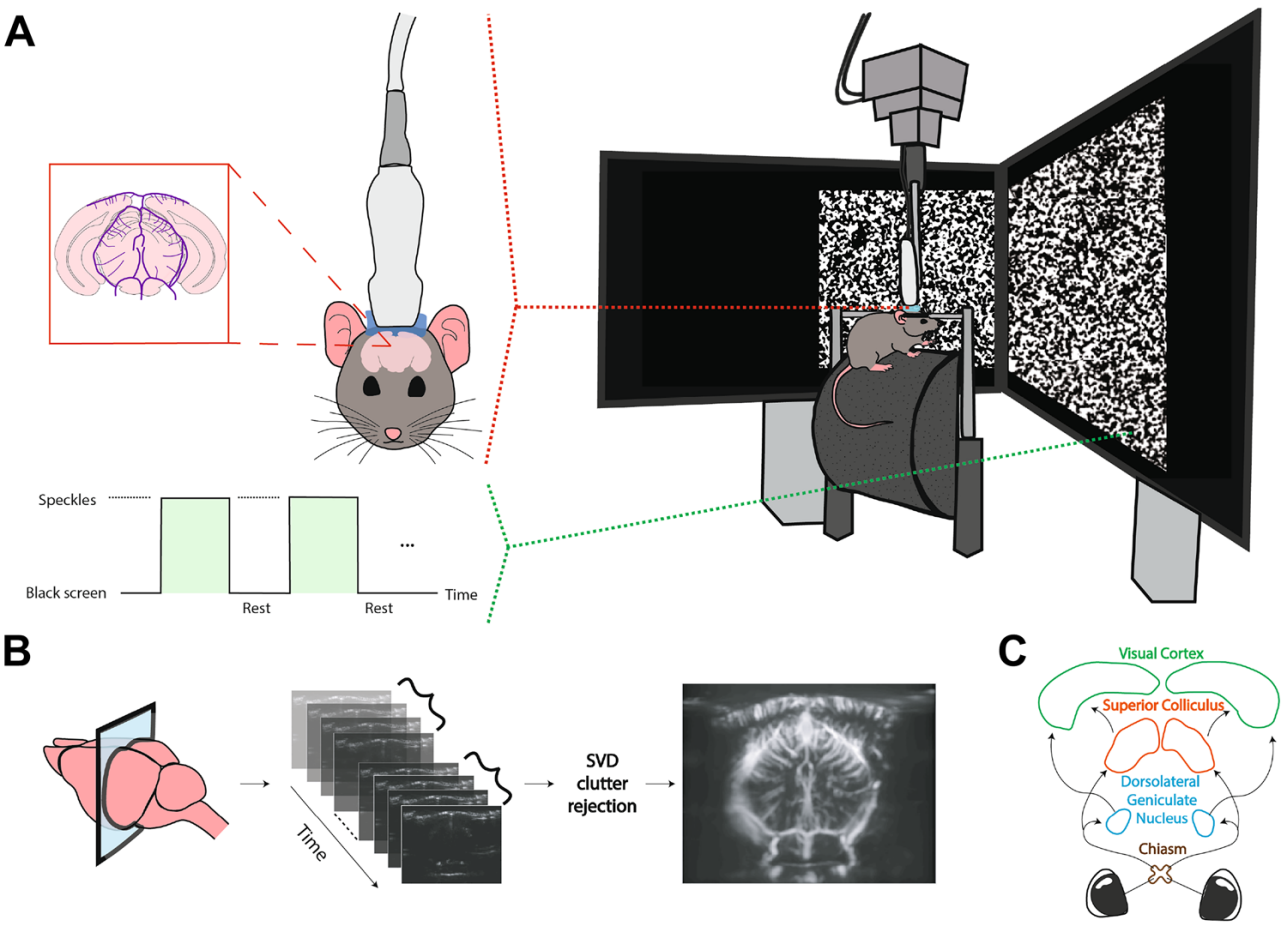
The setup and flowchart for fUS imaging of the ROIs. The setup is shown in A, with the awake, head-fixed mouse walking on a movable wheel. During an experiment, either a visual stimulus (here the speckles) or an entirely black screen (rest) is displayed across both monitors. In B, the process of forming a PDI is demonstrated for a coronal slice. First, backscattered ultrasonic waves obtained at different imaging angles are beamformed, resulting in compound images. Next, the compound images are progressed to SVD clutter filtering in batches to remove the tissue motion from the vascular signal. From each batch, a PDI is constructed by computing the power per-pixel. In C, the ROIs that we will focus on in the rest of this paper are shown. The pointed arrows represent the signal flow for processing of visual information

Before experimental acquisition, a high-resolution anatomical registration scan was made of the exposed brain’s microvasculature so as to locate the most ideal imaging location for capturing the ROIs aided by the Allen Mouse Brain Atlas (Allen Institute for Brain Science, 2015). For data acquisition, 20 tilted plane waves were continuously transmitted from an ultrasonic transducer (Vermon L$22-14$v, 15 MHz) at 800 Hz, which was coupled to the the mouse’s cranial window with ultrasound transmission gel (Aquasonic). A compound image was obtained by Fourier-domain beamforming and angular compounding, and non-overlapping ensembles were formed by concatenating 200 consecutive compound images. Next, we applied SVD-filtering to denoise the images and separate the blood signal from stationary and slow-changing ultrasound signals arising from other brain tissue. More specifically, SVD-filtering was performed on each ensemble by setting the first (i.e., largest) 30% and the last (i.e., smallest) 1% of the singular values to 0, the former rejecting tissue clutter (Demené et al., 2015b) whereas the latter removing noise (Song et al., 2017). Afterwards, the vascular signal of interest was reconstructed back from the remaining singular components (Demené et al., 2015a). Images were upsampled in the spatial frequency domain to an isotropic resolution of $25\mu$m, matching to that of the Allen Reference Atlas. Lastly, a Power-Doppler Image (PDI) was obtained by computing for every pixel the power of the SVD-filtered signal over the frames within the ensemble, providing a final sampling rate of 4 Hz for the PDIs. The time-series of a pixel (Eq. 4) corresponds to the variation of its power across the PDI stream.

For the selection of ROIs, the experimental data was first parcellated using spatial ICA with 10 components (Sala-Llonch et al., 2019). The components of interest were thresholded to reveal a spatial binary mask standing for an anatomical ROI. To obtain a representative time-series for each ROI, we averaged the time-series of pixels which are captured within the boundaries of the corresponding spatial mask. Finally, the ROI time-series were normalized to zero-mean and unit-variance before proceeding with the BTD.

## Results

To demonstrate the power of our BTD-based deconvolution approach, the following sections discuss a simulation study and the results of the *in vivo* mouse experiments respectively.

### Numerical Simulations

We simulated three ROI time-series at a sampling rate of 2 Hz, where each time-series was characterized with a unique HRF, i.e., with a different parameter set $\varvec{\theta }$ (Eq. 9). We assumed that there are two common source signals that build up to the ROI time-series. The first source signal is a binary vector representing the EP. The EP involves 20 repetitions of a 4-seconds stimulus (where the vector takes the value 1) interleaved with $10-15$ seconds of random non-stimulus intervals (where the vector becomes 0). This is the same paradigm that will be used later for deconvolution of the first *in vivo* fUS experiment (“Single Stimulus”). The EP is assumed to drive the hemodynamic activity in all ROIs, but the measured fUS signals are linked to the EP through possibly different HRFs. The second source signal stands for the artifact component and is generated as a Gaussian process with changing mean, in accordance with the system noise and artifacts modeled in (Correa et al., 2005).

**Fig. 5:**
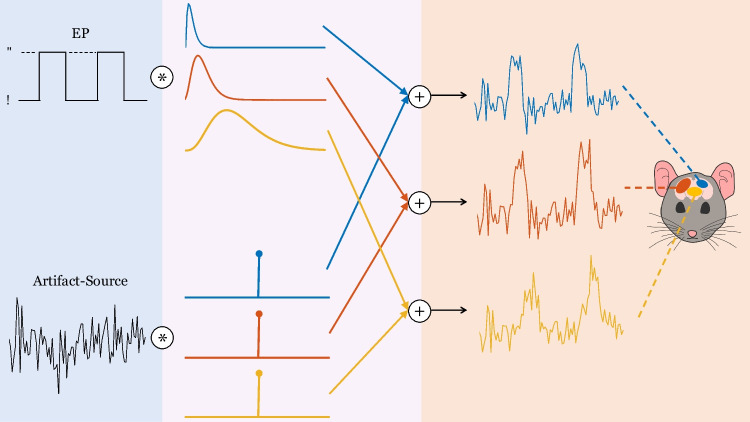
Illustration of the simulator. Both of the simulated sources are shown in the left section, one being task-related (the EP) and one being artifact-related. In the middle section, the convolutive mixing filters are depicted. The filters which are convolved with the EP are the HRFs, whereas the filters which are convolved with the artifact source only differ by their scaling and modeled as impulses, such that their convolution with the artifact source lead to a direct summation on the measured time-series. In the last section, the convolved results are added together to deliver the time-series at each ROI

Each ROI time-series is obtained by convolving the corresponding HRF and the EP, and subsequently adding on the noise source, whose variance is dependent on the region. In addition, the noise variance values are adjusted in order to assess the performance of the proposed method under various signal-to-noise ratios (SNRs). The data generation steps are illustrated in Fig. 5.

We normalized the time-series to zero-mean and unit-variance before proceeding with the BTD. While solving the BTD, we assumed that there was one task-source of interest and one artifact source. We performed a Monte Carlo simulation of 100 iterations for different SNR values. In each iteration, the HRF parameters were generated randomly such that the HRF peak amplitude was random in the range [0, 1], whereas the peak latency (PL, also referred as the rise time or time-to-peak) and width (measured as full-width at half-maximum; FWHM) of the simulated HRFs varied between [0.25, 4.5] and [0.5, 4.5] seconds respectively. These ranges generously cover the CBV-based HRF peak latencies (reported as $2.1 \pm 0.3$ s in (Nunez-Elizalde et al., 2022), and between 0.9 and 2 seconds in (Aydin et al., 2020; Winder et al., 2017; Aydin et al., 2021)) and FWHMs (reported as $2.9 \pm 0.6$ s in (Nunez-Elizalde et al., 2022)) observed in previous mouse studies.

Finally, we defined the following metrics at each Monte Carlo iteration to validate the performance of the algorithm:

- The Pearson Correlation Coefficient between the true EP and the estimated source signal, and
- The absolute PL difference (in terms of seconds) between the true and estimated HRFs, averaged for $M=3$ ROIs.

**Fig. 6:**
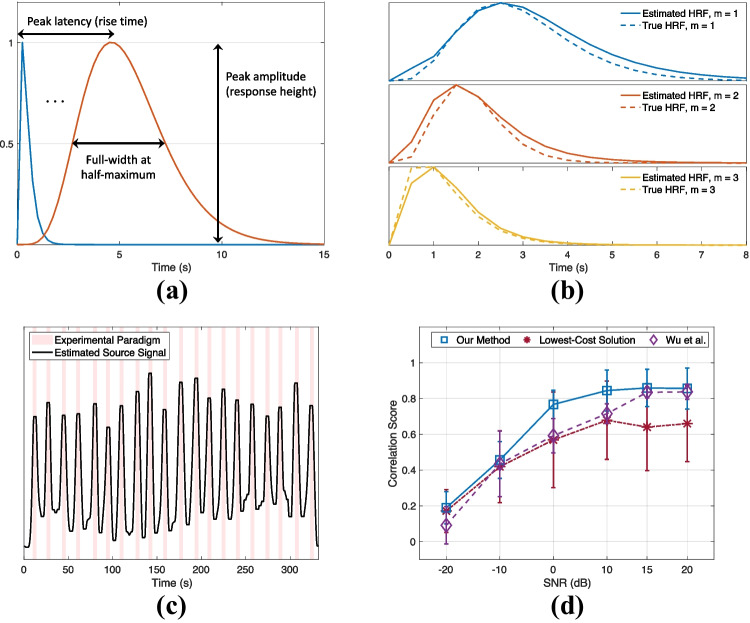
Simulation results

Simulation results are provided in Fig. 6. Under 0 dB SNR, the estimated HRFs have an error of $0.3 \pm 0.4$ (median ± standard deviation) seconds in the peak latencies across the Monte-Carlo iterations. In addition, we compared our EP estimation performance to: *(i)* When the BTD solution providing the lowest cost value is picked, as opposed to the selection procedure proposed in “Identifying a Stable Solution for BTD”, and *(ii)* The method proposed by (Wu et al., 2013). Note that as the method by (Wu et al., 2013) is univariate, we computed their average source signal estimate from all the regions for calculating the correlation score with respect to the simulated EP). The results (Fig. 6d) highlight that the clustering approach proposed for converging to a final solution with BTD yields to a significant increase in the correlation values compared to the lowest-cost solution. The method by (Wu et al., 2013) performs close to our method at high SNR values (15 to 20 dB), yet, their performance significantly deteriorates as the noise power is increased. In the context of real neuroimaging data, this difference could cause a misinterpretation of the underlying source signals and neurovascular dynamics.

**Fig. 7:**
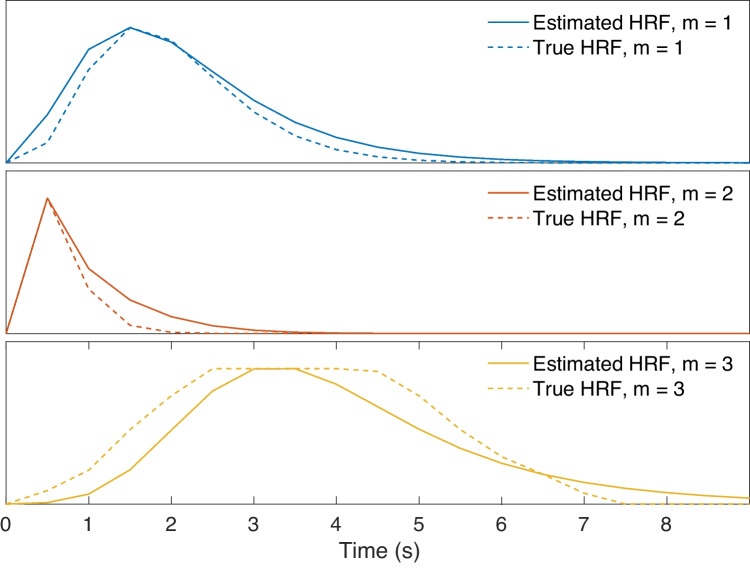
Estimated HRFs in case of an outlier HRF shape (bottom plot), under 0 dB SNR

Lastly, we generated an HRF that is not characterizable by the gamma-model given in Eq. 9 to see how the proposed method will handle an outlier. In this particular case, we assumed that the HRF shape exhibits a plateau, corresponding to a sustained peak response along time, as shown in the bottom plot of Fig. 7. We observed that the proposed method tries to approximate the HRF in the best way possible, while the estimated source still achieves a correlation of 0.72 with the EP under 0 dB SNR. This is slightly less than the mean correlation score (0.77) at this SNR (Fig. 6d). For the same outlier scenario, the source estimate by (Wu et al., 2013) has a correlation of 0.62 with the EP.

### Experimental Data

#### Single Stimulus

**Fig. 8:**
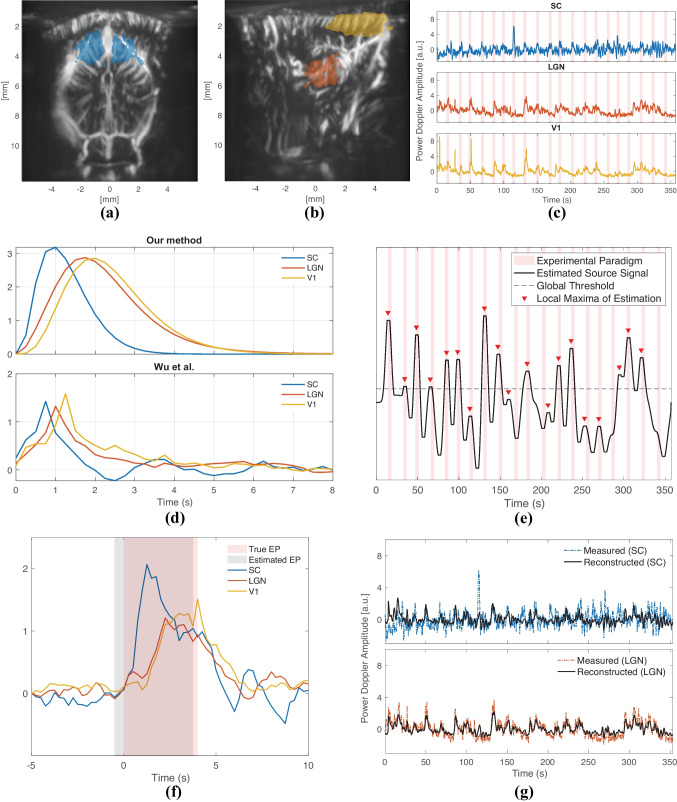
Deconvolution results of the first fUS experiment

In this experiment, we imaged the mouse brain at two slices (one coronal at Bregma $-3.80$ mm, and one sagittal at Bregma $-2.15$ mm (Franklin & Paxinos, 2001)) to capture the ROIs that we wished to analyze: SC, LGN and the primary visual cortex (V1). We first applied ICA to select groups of pixels that involve these ROIs as demonstrated in Fig. 8a and b, showing SC in the former (coronal slice); LGN and V1 in the latter plot (sagittal slice). Note that, we needed to image two different slices in order to achieve a good capturing of all the ROIs. Although two of them are presented here, we actually imaged more slices with the same experimental paradigm so as to select the best location for the ROIs. We investigated the time-series of the different slices and established that the responses within the same ROI were reproducible across runs. Hence, we concluded that proceeding with jointly decomposing the ROI time-series acquired from different slices is a valid approach.

The raw, normalized fUS time-series belonging to each ROI are displayed in Fig. 8c. By deconvolving this multivariate time-series data, we estimated the region-specific HRFs and the underlying source signal of interest.

We compared our deconvolution results to those by (Wu et al., 2013). According to the HRFs estimated by our method (Fig. 8d, top plot), LGN and V1 have a clear lag in time compared to SC. On the other hand, the HRFs estimated by (Wu et al., 2013) ((Fig. 8d, bottom plot) are observed to be following each other more closely. The ordering of the HRFs is the same in both methods. The difference between the predicted HRFs by two methods likely arises from the fact that (Wu et al., 2013) estimates a different source signal for each ROI. In other words, as their input signals are assumed different, the impulse responses of each ROI can as well be different compared to when a single common source is assumed, as by our method.

More specifically, (Wu et al., 2013) offers three source signals for three ROIs, and the source signal in SC is highly aligned with the EP with a correlation coefficient of 0.57, whereas the correlation drops to 0.4 in V1 and 0.16 in LGN. The estimated source signal by our method is assumed to evoke the responses of all ROIs, and has a correlation coefficient of 0.5 with the EP. The low correlation of the source signal in LGN estimated by (Wu et al., 2013) makes it challenging to decipher its HRF, such as for understanding how swiftly it reacts to the visual stimulus compared to SC or V1. Although a univariate approach might be advantageous in certain situations, when dealing with ROIs that are known to be alerted by the same external or internal stimulus, the presumption that they share a common input signal not only intuitively makes sense, but also makes the interpretation of the HRFs easier. On the other hand, our method aims at finding the best-fitting transfer function between estimated stimulus events and the measurements. This way, the estimated HRFs provide an insight into how fast (by the peak latency) or how long (by the width) a region reacts to a common triggering event. The HRFs estimated by our method point to a peak latency of 1 s in SC, 1.75 s in LGN and 2 s in V1. Similarly, the FWHMs are found as 1.25 s in SC, 1.75 s in LGN and 1.75 s in V1. These results manifest that SC gives the fastest reaction to the visual stimulus amongst the ROIs, followed by the LGN. In addition, the HRF in SC is observed to be steeper than in LGN and V1.

Figure 8e demonstrates the source signal estimated by the proposed method. Unlike the simulations, we see that the source signal exhibits a substantial variation in amplitude across time. In order to interpret this behavior of the estimated source signal, we further investigated the raw fUS signals shown in Fig. 8c. When the responses given to consecutive repetitions of the stimulus are compared within each region, it can be observed that SC reacts most consistently to the stimulus, while the reproducibility of the evoked responses in LGN and V1 (particularly in V1) are much lower, especially in the second half of the repetitions. To better quantify and compare the region-specific differences in response-variability, we computed the Fano factor (FF) as the ratio of the variance to mean peak amplitude of each region’s post-stimulus response (Rahnev et al., 2012), defined in a window [0, 10] seconds after a stimulus has been shown. We found an FF value of 0.23, 0.42 and 0.8 respectively for SC, LGN and V1. These findings indicate that the consistency of the hemodynamic response strength is halved from SC to LGN, and again from LGN to V1.

There are cases where there is a very subtle reaction (as detected by fUS) to the stimulus in V1, such as in repetitions 10, 12, 15, 16 and 20. These repetitions coincide with the points in Fig. 8e wherein the most considerable drops in the estimated source signal were observed. As such, the variability of responses can explain the unexpected amplitude shifts of the estimated source signal.

Due to its changing amplitude, binarizing the estimated source signal using a single global threshold would not work well (Fig. 8e) for the sake of discovering the exact on- and off- times of the stimulus as found by our method. However, it is still possible to observe local peaks of the estimated source signal occurring around the times that the stimulus was shown. While applying a global threshold can uncover 13 out of 20 repetitions, with a detection of local peaks, this number increases to 19 out of 20 repetitions. After detecting the peaks, we located the time points where for the first time a significant rise (and drop) was observed before (and after) the peak, leading to the starting (and ending) times of the estimated repetitions. Hence, we obtained an estimation of the EP by constructing a binary vector of all 0’s with the exception of the time periods in between the predicted starting and ending points. In Fig. 8f, we compared our EP estimation (averaged across repetitions) with the true EP. We can appreciate that our EP estimation is a slightly shifted ($<0.5$ seconds) version of the true EP. In this figure, we also displayed the repetition-averaged responses in SC, LGN and V1; which as well support the HRFs found by our method- with the SC response preceding LGN and V1 by a relatively large separation in time.

Note that the observed trial-by-trial variability in temporal profile across the measured HRs underlines the importance of estimating the source signal. The conventional definition of the EP strictly assumes that the input of the convolution leading to the neuroimaging data (Eq. 1) is the same ($=1$) at each repetition of the stimulus. This would mean that the exact same input, shown at different times, outputs different responses, which would evidence a dynamic system (Friston et al., 2000; Friston et al., 2003). However, estimating the source signal allows for a non-binary and flexible characterization of the input, and thus LTI modelling *can* remain plausible. Although extensive analysis of the repetition-dependent behavior of the vascular signal is beyond the scope of this work, we will mention its possible foundations in Discussions.

**Fig. 9:**
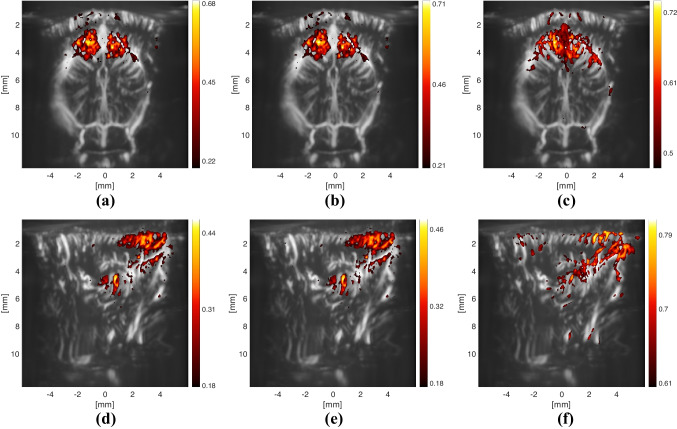
Correlation images obtained by correlating the pixel time-series with a different signal at each plot. All of the correlation images are thresholded such that only the pixels with a significant correlation value (with a z-score $\ge 2$) are displayed. The colorbars denote the value of the Pearson correlation coefficient, and they are scaled up to the maximum value achieved with the corresponding approaches. Note that the approach used in (**c**) and (**f**) leads to a similar result as using SC and LGN respectively as a seed region, since the result of the convolution matches well to the average ROI responses, as shown in Fig. 8g

Lastly, we explored how the estimated source signal and HRFs can be used to generate different correlation images of the brain (Fig. 9). Although the active regions do not change between using an optimally-delayed version of the EP ((a) or (d)) or an HRF ((b) or (f)), the maximum correlation value increases slightly when the HRF is utilized. For (a) and (d), the EP was delayed by the value, between 0 to 10 seconds, that provided the highest overall correlation, measured by the mean of non-negative correlations. The optimal delay for the coronal slice (containing the SC) was 0.75 seconds (which is 0.25 s less than the peak latency of the estimated HRF in SC), whereas the one in the sagittal slice was found as 1.75 seconds (which is equal to the peak latency of the estimated HRF in LGN).

#### Multiple Stimuli

In this experiment, we imaged the mouse brain at Bregma $-3.52$ mm, and defined 5 different stimulus conditions based on the location where the grating stimulus was presented: Leftmost (LM), Slight-Left (SL), Front (F), Slight-Right (SR), and Rightmost (RM). In this experiment our aim was to demonstrate that the proposed method is able to separate multiple underlying sources. More specifically, we assume that the 5 different stimulus locations will evoke hemodynamic activity at different areas within the brain. Therefore, we expect that we can recover the timings of the different stimulus locations as different sources in our model (Eq. 12).

**Fig. 10:**
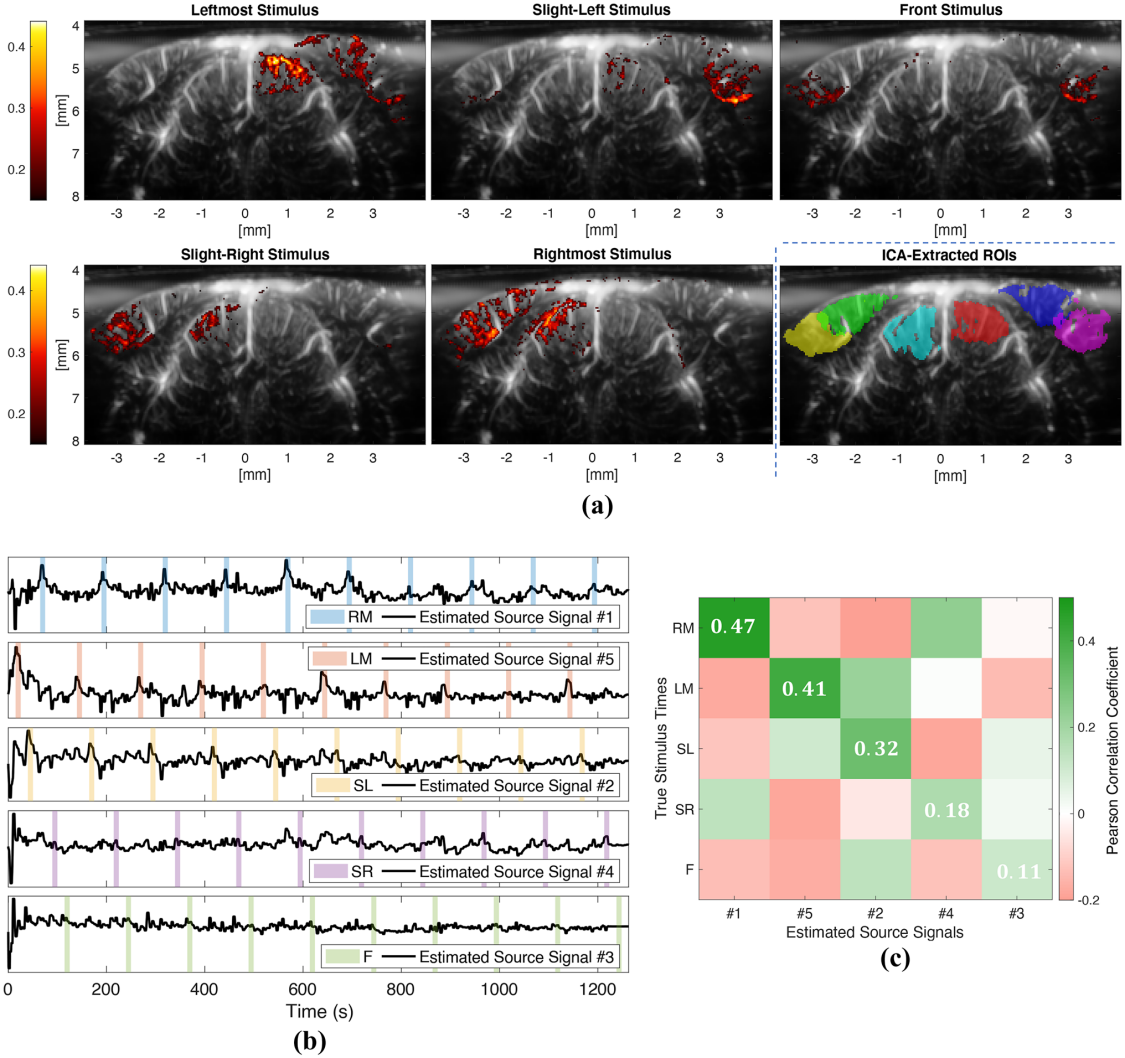
Results of the multiple-stimuli experiment

We again started by selecting the ROIs by applying ICA. As it can be seen in Fig. 10a, ICA is able to extract all the regions exhibiting a significant correlation with the various stimulus conditions. Next, we ran BTD on the displayed regions while assuming five task-sources and one artifact-source. The estimated source signals are shown in Fig.10b, which reveals that each estimated source signal tends to fit to the timings of one particular stimulus condition, while suppressing the rest of the stimulus conditions. In order to visualize this more clearly, we plotted the estimated sources against the stimulus condition they designate.

For a more precise evaluation of the proposed method’s performance in this scenario, we computed the matrix given in Fig. 10c, where each column shows the correlation values of one estimated source signal with all the stimulus conditions. Note that, an ideal version of this matrix would correspond to an identity matrix (i.e., it would have 1’s at the diagonal and 0’s at the non-diagonal entries), meaning that each source is matched with the timings of exactly one stimulus location. In other words, as important it is to detect the timings of a stimulus condition via an estimated source (which would be assured by 1’s at the diagonal), a perfect separation of sources would also imply that no two or more stimulus conditions exist simultaneously within one estimated source signal (which would be assured by 0’s at the non-diagonal terms). For example, our source estimation results show that the stimuli on the right screen (RM and SR) are more prone to be confused for one another, which is rather understandable, as the correlation images (Fig. 10a) suggest that the same regions can react similarly to both conditions, making it challenging to discriminate between the two (the same is valid for the stimuli on the left screen as well).

Furthermore, the conditions that were identified most accurately are RM and LM, which are also the ones that provide the best overall correlation images. Similarly, the condition that was identified most poorly, the front location, is also the one that has the lowest correlation with the stimulus. These observations confirm that the more the brain reacts to a stimulus, the better that stimulus can be recovered with the proposed deconvolution approach, and the more regions differ in their reaction to two stimulus conditions, the easier our method can distinguish between them. Last but not least, we compared our source estimation results with (Wu et al., 2013) in Table 1. Our method achieves a better score in both identifying the stimulus conditions, and their separation from each other.

**Table 1:** Results of the multiple-stimuli experiment. We first identified the source signal which achieves the highest correlation with a stimulus condition (ideally, 1). Next, we evaluated how much this source signal correlates with the other stimulus conditions (ideally, 0) by summing up these correlation values. Note that, at this step, we considered negative correlation values as 0. We compared our results to those by (Wu et al., 2013), and the best result achieved by either method is marked as bold for each stimulus condition

Stimulus Location	Highest Correlation		False Correlations	
Stimulus Location	(Wu et al., 2013)	Ours	(Wu et al., 2013)	Ours
RM	0.34	$\mathbf {0.47}$	$\mathbf {0.07}$	0.14
LM	$\mathbf {0.49}$	0.41	0.18	$\mathbf {0.1}$
SL	0.31	$\mathbf {0.32}$	$\mathbf {0.19}$	0.35
SR	$\mathbf {0.21}$	0.18	0.33	$\mathbf {0.25}$
F	$\mathbf {0.12}$	0.11	0.4	$\mathbf {0.08}$
Mean Score	0.29	$\mathbf {0.3}$	0.23	$\mathbf {0.18}$

The proposed method estimates HRFs that are source-specific as well as region-specific. As the rightmost stimulus was extracted the most accurately, we looked at the HRFs of the first (i.e., top plot of Fig. 10b) estimated source. We again observed a delay in the peak latency of the HRF in V1, which peaks at 1.1 s, compared to that of SC, which peaks at 0.8 s (HRF estimates not shown). The difference between the HRF peaks is not as high as in the first experiment, which is also the case for the repetition-averaged fUS responses of the ROIs. This could be the result of displaying different types of visual stimulus in the experiments (sinusodial grating versus speckles), as the HRF is shown to exhibit stimulus-dependent behavior (Lewis et al., 2018; Aydin et al., 2020).

## Discussion

In this study, we considered the problem of deconvolving multivariate fUS time-series by assuming that the HRFs are parametric and source signals are uncorrelated. We formulated this problem as a block-term decomposition, which jointly estimates the underlying source signals and region-specific HRFs. In other words, the proposed method for deconvolution of the hemodynamic response has the advantage of not requiring the source signal(s) nor the HRFs to be specified. As such, it can take into account regional differences of the HRF, as well as recover numerous sources besides the EP, that are unrelated to the intended task and/or outside of the experimenters’ control. We investigated the fUS response in several regions of the mouse brain, namely the SC, LGN and the visual cortex, which together compose significant pathways between the eye and the brain.

We tested our method in two *in vivo* fUS experiments. In the first experiment, we assumed one common task-source for the ROIs (SC, LGN and V1) and inspected the estimated source signal and HRFs. It is important to mention that the responses of the selected ROIs are shown to be modulated by the same stimulus in prior works (Ahmadlou et al., 2018; Gesnik et al., 2017; Lewis et al., 2018), as can also be seen via the stimulus-correlation maps of our experiment (Fig. 9) which reveal significant values in these ROIs. In the estimated source signal, we observed unforeseen amplitude variations along time. To better understand this behavior, we investigated the hemodynamic responses in the selected ROIs across repetitions. We noticed that the response variability in the visual system increases from the subcortical to the cortical level. Consistent with our findings, electrophysiological studies such as (Kara et al., 2000) report an increase in trial-by-trial variability from subcortex to cortex, doubling from retina to LGN and again from LGN to visual cortex. Variability in responses could be related to external stimulation other than the EP, such as unintended auditory stimulation from experimental surroundings (Ito et al., 2021). In addition, literature points to eye movements as a source of high response variability in V1, a behavior which can be found in head-fixated, but awake mice following attempted head rotation (Meyer et al., 2020), which can extraordinarily alter stimulus-evoked responses (Gur & Snodderly, 1997).

Based on the estimated HRFs, we noted that SC has the fastest reaction to stimuli, followed respectively by LGN and V1. As V1 does not receive direct input from the retina, but via LGN and SC, its delayed HRF is consistent with the underlying subcortical-cortical connections of the visual processing pathway, as has also been reported by (Brunner et al., 2021; Lau et al., 2011; Lewis et al., 2018). What’s more, the SC’s particular aptness to swiftly respond to the visual stimulus aligns with its biological function to indicate potential threats (such as flashing, moving or looming spots (Gale & Murphy, 2016; Wang et al., 2010; Inayat et al., 2015)).

In the second experiment, we explored the source-detection capabilities of our method further in a more complex setting, with five different stimulus conditions determined by five different locations that the stimulus was presented. The proposed method was able to successfully detect the timings of these locations and differentiate them from one another, particularly those that elicited a stronger and a more discriminative response in the mouse brain.

For both experiments, we considered the number of sources to be known, which is usually not the case in practice. In order to investigate the sensitivity of our approach to this choice, we tried selecting less or more sources in the second fUS experiment. Selecting less number of sources (3 sources) resulted in similar stimuli being grouped together under one source signal. Particularly, one of the estimated sources revealed the timings of both the leftmost and slight-left stimuli, whereas another estimated source reflected both the rightmost and slight-right stimuli. On the other hand, when the number of sources was overestimated, we observed that the estimated sources were more interleaved with one another, resulting in slightly smaller correlation values with the true stimulus times (at least while using the same set of parameters). Overall, we conclude that a sub-optimal choice for the number of sources still leads to reasonable results. Nevertheless, a possible future extension of this work would be to explore methods for estimating the number of sources, such as information criterion-based approaches (Bai & He, 2006).

Compared to our previous BTD-based deconvolution, we have made several improvements in this work. To start with, the current method exploits all the structures in the decomposition scheme. In particular, previously the core tensor representing the lagged source autocorrelations was structured to be having Toeplitz slices, yet, these slices were not enforced to be lagged versions of each other. Incorporating such theoretically-supported structures significantly reduced the computation time of BTD by lowering the number of parameters to be estimated. In addition, we increased the robustness of our algorithm by applying a clustering-based selection of the final HRF estimates amongst multiple randomly-initialized BTD runs. Nevertheless, the formulated optimization problem is highly non-convex with many local minima, and the simulations show that there is still room for improvement. For instance, different clustering criteria can be applied for choosing the “best” solution (Himberg et al., 2004). Furthermore, there are several hyperparameters - namely the HRF filter length, number of time lags in the autocorrelation tensor, and the number of BTD repetitions - which affect the algorithm’s performance as well as convergence speed. For instance, although the run-time of one BTD is not very long, repeating it 20 times to converge to a better solution, as done in our current setting, considerably increases the overall time. Instead, it is possible to search for different initialization strategies and run fewer BTDs. Note that the computational complexity of BTD is linear in the number of sources, in the order of the tensor and in the product of the sizes of each mode (Sorber et al., 2013b). The autocorrelation tensor in our solution is of size $ML'\times ML'\times K$, which means that the computational complexity increases quadratically with the number of ROIs (*M*) and the number of variables used for calculating the autocorrelations ($L'$). For a higher number of *M*, the complexity level can be preserved by lowering $L'$, which could result in a loss of accuracy. In that case, the trade-off between complexity and accuracy should be thoroughly analyzed. All in all, our purpose in this study was to show that the convolutive mixtures modelling and the BTD formulation together offer a deconvolution framework that is able to accommodate and unveil many unknowns regarding hemodynamic activity, yet there is more to discover by conducting more real-life experiments for fully acknowledging the potential of the proposed method.

## Conclusion

In this paper, we deconvolved the fUS-based hemodynamic response in several regions of interest along the mouse visual pathway. We started with a multivariate model of fUS time-series using convolutive mixtures, which allowed us to define region-specific HRFs and multiple underlying source signals. By assuming that the source signals are uncorrelated, we formulated the blind deconvolution problem into a block-term decomposition of the lagged autocorrelation tensor of fUS measurements. The HRFs estimated in SC, LGN and V1 are consistent with the literature and align with the commonly accepted neuroanatomical functions and interconnections of said areas. In the meantime, the estimated source signals, whether a single task or multiple tasks were employed throughout the experiment, can be identified successfully in terms of the timings they were presented. Overall, our results show that convolutive mixtures with the accompanying tensor-based solution provides a flexible framework for deconvolution by revealing an elaborate characterization of hemodynamic responses in functional neuroimaging data.

## Information Sharing Statement

The data and MATLAB scripts that support the findings of this study are publicly available at https://github.com/ayerol/btd_deconv.
