# Correction to: Deconvolution of the Functional Ultrasound Response in the Mouse Visual Pathway Using Block-Term Decomposition

**DOI:** 10.1007/s12021-022-09619-x

**Published:** 2022-12-26

**Authors:** Aybüke Erol, Chagajeg Soloukey, Bastian Generowicz, Nikki van Dorp, Sebastiaan Koekkoek, Pieter Kruizinga, Borbála Hunyadi

**Affiliations:** 1 Circuits and Systems (CAS), Department of Microelectronics, Delft University of Technology, Mekelweg 5, Delft, 2628 CD The Netherlands; 2 Center for Ultrasound and Brain Imaging at Erasmus MC (CUBE), Department of Neuroscience, Erasmus Medical Center, Doctor Molewaterplein 40, Rotterdam, 3015 GD The Netherlands

**Correction to: Neuroinformatics**

10.1007/s12021-022-09613-3

The original version of this article was revised to update the Figure 8 (panel B) image. The correct image should have a yellow shape as presented below.
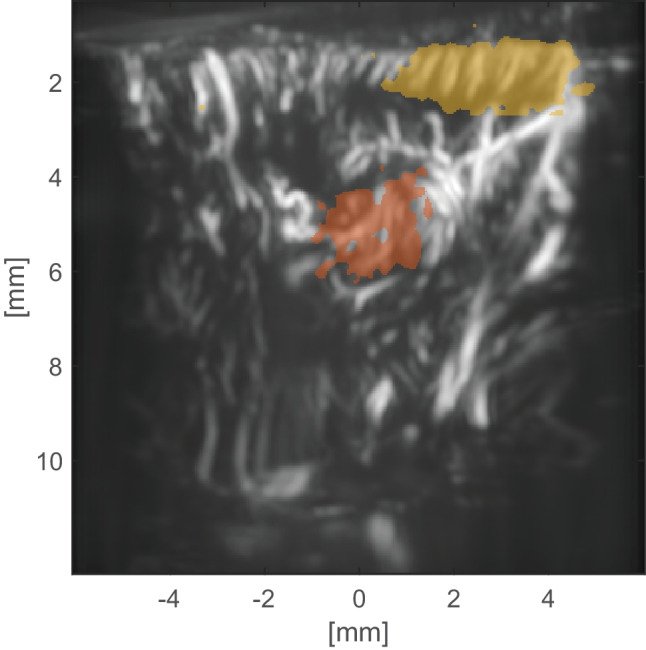


The original article has been corrected.

